# Structural and Functional Determinants of AC8 Trafficking, Targeting and Responsiveness in Lipid Raft Microdomains

**DOI:** 10.1007/s00232-019-00060-x

**Published:** 2019-02-12

**Authors:** Valentina G. Tabbasum, Dermot M. F. Cooper

**Affiliations:** Department of Pharmacology, University of Cambridge, Tennis Court Rd., Cambridge, CB2 1PD UK

**Keywords:** Adenylyl cyclase, cAMP, Caveolin, Lipid rafts, Plasma membrane, Cholesterol

## Abstract

The fidelity of cAMP in controlling numerous cellular functions rests crucially on the precise organization of cAMP microdomains that are sustained by the scaffolding properties of adenylyl cyclase. Earlier studies suggested that AC8 enriches in lipid rafts where it interacts with cytoskeletal elements. However, these are not stable structures and little is known about the dynamics of AC8 secretion and its interactions. The present study addresses the role of the cytoskeleton in maintaining the AC8 microenvironment, particularly in the context of the trafficking route of AC8 and its interaction with caveolin1. Here, biochemical and live-cell imaging approaches expose a complex, dynamic interaction between AC8 and caveolin1 that affects AC8 processing, targeting and responsiveness in plasma membrane lipid rafts. Site-directed mutagenesis and pharmacological approaches reveal that AC8 is processed with complex N-glycans and associates with lipid rafts en route to the plasma membrane. A dynamic picture emerges of the trafficking and interactions of AC8 while travelling to the plasma membrane, which are key to the organization of the AC8 microdomain.

**Electronic supplementary material:**

The online version of this article (10.1007/s00232-019-00060-x) contains supplementary material, which is available to authorized users.

## Introduction

The residence of adenylyl cyclases (ACs) at the plasma membrane is a central premise of cAMP signalling, although their presence there is much understudied. The ever-expanding complexity of intracellular protein trafficking pathways is being elucidated from growing insights on biosynthesis, post-translational processing and molecular assembly of membrane proteins and their vesicular trafficking and endocytosis (Bonifacino and Glick 2004; Doherty and McMahon 2009; Vagin et al. 2009; Parton and Howes 2010; Feige and Hendershot 2013). However, little is known about trafficking routes of ACs and their targeting and compartmentalization to specific plasma membrane microdomains (Gu et al. 2001, 2002; Swaney et al. 2006; Cooper and Tabbasum 2014, Johnstone et al. 2018).

Nine membrane-bound (1–9) and a soluble AC (10) have been cloned and characterized (Cooper 2003). Among these species, adenylyl cyclase 8 (AC8) which is confined to neuronal and secretory tissue has been implicated in long-term memory and long-term potentiation (Cali et al. 1994; Ferguson and Storm 2004; Conti et al. 2007). AC8, which is activated by calcium (Ca^2+^) acting via calmodulin (CaM), in intact cells displays a specific dependence on Ca^2+^ entering the cell through store-operated Ca^2+^-entry (SOCE) processes in non-excitable cells (Fagan et al. 1996). The SOCE process involves translocation of the endoplasmic reticulum (ER) Ca^2+^ sensor, stromal interaction molecule 1 (STIM1) upon depletion of Ca^2+^ stores to the plasma membrane, where it binds to and activates the channel component, Orai1, which permits an inward extracellular Ca^2+^ (SOCE) current (Parekh and Putney 2005) that activates AC8. The strict dependence of AC8 on regulation by SOCE relies on direct binding of its N-terminus (AC8-Nt) to the N-terminus of Orai1, which resides, along with AC8, in the so-called lipid raft domains of the plasma membrane (Willoughby et al. 2012).

In their natural state, biological membranes are dynamically heterogeneous, both in terms of their chemical composition and physical properties. Lipid rafts are envisaged as dynamic assemblies of cholesterol and sphingolipids in the plane of the membrane (Brown and London 1998; Simons and Vaz 2004; Sezgin et al. 2017). The lipid and protein composition of rafts has consequences for their biophysical as well as biochemical properties that differ from non-raft domains. Lipid rafts are difficult to detect in live cells except with the use of labelled probes by elegant biophysical methods and modern microscopic techniques. Detailed exploration of the nature of rafts and their role in trafficking signalling molecules—particularly through a range of cellular compartments where they undergo covalent modifications—is not readily accessible (Lagerholm et al. 2005; Levental and Veatch 2016; Sezgin et al. 2017). In order to address these issues, a combination of biochemical and live-cell methods combined with rigorous statistical assessments of the consequences of pharmacological manipulations and for the responsiveness of the adenylyl cyclase to physiological regulation must be adopted. That strategy is adopted in the current investigation. The fluctuating nature and nanometer scale of lipid rafts, taxes the isolation of uncontaminated lipid raft microdomains for proteomics analysis (Mohamed et al. 2018). Nevertheless, in the last decade, a considerable number of studies using an array of biochemical extraction methods in reporting the proteomic analysis of bulk lipid rafts prepared from diverse cells and tissues have arrived at a reasonable consensus of raft-resident proteins [A searchable database for mammalian lipid raft proteomics data (RaftProt) has been developed (Shah et al. 2015). Of note, within the present context, both AC8 and Orai1 are encountered in that database.].

Caveolae are transient platforms that scaffold and compartmentalize characteristic proteins to initiate diverse molecular signalling routes (Cohen et al. 2004). The scaffolding properties of caveolins residing in caveolae are generally mediated by the so-called caveolin scaffolding domain (CSD). This 20 amino-acid stretch located at the Nt of caveolins binds to and often suppresses the downstream signalling of caveolin-interacting molecules (Razani et al. 2002).

Soluble, intracellular pools of caveolin have been identified in various subcellular compartments, which suggests a role of caveolae in intracellular trafficking (Kurzchalia et al. 1992; Dupree et al. 1993; Gagescu et al. 2000; Pol et al. 2005). Indeed, caveolae permit intracellular trafficking of intra- and extracellular elements through biosynthetic, transcytotic and endocytotic routes (Bastiani and Parton 2010).

The sophisticated organization of AC8 and its partitioning alongside components of cAMP microdomains in lipid rafts is required for its regulation by SOCE. Perturbing the integrity of lipid rafts alters both the basal and stimulated activity of AC8, along with its mobility in the plasma membrane, which underscores the importance of these domains in AC8 function and distribution (Smith et al. 2002; Ayling et al. 2012). Various determinants, such as N-linked glycosylation, cholesterol recognition amino-acid sequence (CRAC) motifs, binding to the cytoskeleton and tethering by un-identified scaffolds, have been envisaged to localize AC8 to these domains (Pagano et al. 2009, Ayling et al. 2012). At the same time, limited information exists regarding the biosynthesis, post-translational processing and trafficking routes of AC8 (Gu et al. 2001; Crossthwaite et al. 2005; Cooper and Crossthwaite 2006).

Consequently, in the present study, we investigate the mechanisms which traffic, target and segregate AC8 to lipid rafts, alongside the ramifications of such events on AC8 responsiveness. A potential association of AC8 with caveolin1 is investigated, as well as re-addressing the role of N-linked glycosylation of AC8 and its associations with the actin cytoskeleton, as a possible means by which AC8 is recruited to lipid rafts. By using biochemical, high-resolution microscopy, mutagenesis and pharmacological tools, we suggest that AC8 assembly into lipid raft microdomains is a multi-step process that relies on numerous structural and functional elements, whose complexity has not hitherto been appreciated.

## Experimental Procedures

### Constructs

Epac2-camps was a gift from Martin Lohse (Würzberg University, Germany) (Nikolaev et al. 2004). AC8-HA was created by Dr. Antonio Ciruela by PCR, and this insert was cloned into pcDNA 3.0 between the sites *Kpn*1 and *Xba*1. YFP-AC8 was generated by cloning AC8 cDNA between the Apa1 and *Xba*1 restriction sites of pEYFP-C1. GFP-AC8^N814Q,N818Q,N855E^ (GFP-AC8ΔN-gly) was generated by Pagano et al. (2009).

YFP-AC8^Y144A, F151A, F1144A, Y1146A, Y1151A^ (YFP–AC8Δcav) was generated by Dr. Jessica Sorge by site-directed mutagenesis according to the QuickChange protocol (Stratagene, La Jolla, CA) using the fusion high-fidelity polymerase kit (Finnzymes) according to the manufacturer’s instructions using YFP-AC8 as a template. AC8^Y144A^ was introduced using the forward and reverse primer sequence, 5′-CTCGGATTTCTTCCTCAATGGGGGAGCCAGCGCCCGTGGGGTCATTT-3′ and 5′-AAATGACCCCACGGGCGCTGGCTCCCCCATTGAGGAAGAAATCCGAG − 3′ ,respectively. AC8^F151A^ was introduced using the forward primer sequence, 5′-CAGCGCCCGTGGGGTCATTGCCCCAACCCTA-3′ and reverse, 5′-TAGGGTTGGGGCAATGACCCCACGGGCGCTG-3′. AC8^F1144A^ was introduced using the forward and reverse primer sequence, 5′-GATCAGGGCTTTGCCGCCGACTACCGGGGAGA-3′ and 5′-TCTCCCCGGTAGTCGGCGGCAAAGCCCTGATC-3′. AC8^Y1146A^ was introduced using the forward and reverse primer sequence, 5′- AGGGCTTTGCCGCCGACGCCCGGGGAGAGATATATG-3′ and 5′-CATATATCTCTCCCCGGGCGTCGGCGGCAAAGCCCT-3′. AC8^Y1151A^ was introduced using the forward and reverse primer sequence, 5′-CCGCCGACGCCCGGGGAGAGATAGCTGTGAAGGGCAT-3′ and 5′-ATGCCCTTCACAGCTATCTCTCCCCGGGCGTCGGCGG-3′.

### Cell Culture and Transfections

Stable expression of constructs was achieved as previously described (Delint-Ramirez et al. 2015). For single-cell cAMP measurements, HEK293 cells (European Collection of Cell Culture) were transfected with 0.5 µg of YFP-AC8, YFP-AC8Δcav or GFP-AC8ΔN-gly and 1 µg of Epac2-camps sensor using the Lipofectamine 2000 (Invitrogen) transfection method according to the manufacturer’s instructions. For knock-downs, either caveolin1 small interfering (si) RNA or control siRNA (Santa Cruz Biotechnology) was added at 30 nM final concentration following the manufacturer’s instructions. Real-time measurements of cAMP were performed 48-h post-transfection.

### Single-Cell FRET Measurements

Real-time FRET measurements were performed using an EM CCD Ixon, an EMCCD camera (Andor, Belfast) and an Optosplit (505DC) (Cairn Research) as previously described (Willoughby et al. 2010). The Optosplit divides the CFP (470 nm) and YFP (535 nm) emission images. Cells were excited at 435 nm using a monochromator (Cairn Research) and 51,017 filter set (Chroma Technology Corp) attached to a Nikon eclipse TE2000-S microscope (×40 oil-immersion objective) for dual-emission ratio imaging. Image processing was performed as described previously (Willoughby et al. 2012). Single-cell FRET data were plotted as changes in background subtracted 470 nm versus 535 nm (*CFP*/*YFP*) emission ratio relative to maximum FRET ratio change recorded with saturating cAMP concentrations (maximum; Max) concentration attained by addition of a cocktail of 10 µM Forskolin and 100 µM IBMX.

### Lipid Raft Isolation

Cold detergent extraction of lipid rafts and flotation on sucrose density was carried out as previously described (Delint-Ramirez et al. 2015).

### Immunoblotting

Proteins were resolved using 6, 10 or 12% SDS–polyacrylamide gels as previously described (Delint-Ramirez et al. 2015; Willoughby et al. 2012). Following blocking, membranes were incubated overnight at 4 °C with the following antibodies: anti-caveolin antibody (1:2000, BD Biosciences), anti-GFP antibody (1:10,000, Abcam), anti-β-adaptin antibody (1:5000, Santa Cruz Biotechnology), anti-actin antibody (1:1000, Sigma) and anti-HA (1:5000, Sigma) or anti-GST antibody (1:40,000; Sigma) in TTBS containing 1% (*w*/*v*) skimmed milk.

The membranes were incubated for 1 h with the following secondary antibodies: goat anti-mouse IgG conjugated to horseradish peroxidase (1:20,000, Promega for anti-HA, anti-GST, anti-GFP and anti-actin) or goat anti-rabbit IgG conjugated to horseradish peroxidase (1:20,000, Fisher Scientific, for anti-caveolin and anti-β-adaptin) in TTBS containing 5% (*w*/*v*) skimmed milk. Detection of proteins and quantification of immunoreactive bands were performed as previously described (Delint-Ramirez et al. 2015).

### Cell Treatments and Immunocytochemistry

Cells were plated onto 22-mm poly-l-lysine-coated coverslips and treated with the indicated drugs after 24 h. Where appropriate, cells were incubated with 2 mM Latrunculin B (Lat B; Merck), 10 mM methyl-β-cyclodextrin (MβCD; Sigma) or 5 µg/ml Brefeldin A (BFA; Sigma) for 1 h at 37 °C. Cells were fixed and permeabilized as previously described (Ayling et al. 2012).

For the detection of endogenous caveolin1, cells were incubated with PBS containing 0.5% goat serum; and anti-caveolin antibody (1:1000, BD Biosciences) for 1 h at 37 °C. Coverslips were rinsed three times with PBS for 5 min and incubated with PBS containing 0.5% goat serum and AlexaFluor555-conjugated goat anti-rabbit antibody (1:200, Invitrogen) for 1 h at 37 °C. Coverslips were rinsed and mounted using Hardest Vectashield with DAPI (Vector Laboratories) and stored at 4 °C. Staining of actin filaments was achieved by blocking the coverslips with PBS containing 1% BSA for 30 min at 37 °C and then incubation in PBS containing BSA; 1% and AlexaFluor568-conjugated phalloidin (1 U/ml, Invitrogen) for 2 h at 37 °C. Staining with phalloidin was performed on fixed, permeabilized cells. Visualization of plasma membranes stained with CellMask™ Deep Red (Invitrogen) was performed in live cells. HEK293 cells were incubated with media containing CellMask™ Deep Red (5 µg/µl, Invitrogen) for 10 min at 37 °C. For staining with WGA, cells were incubated with AlexaFluor555-conjugated WGA (5 µg/ml; Invitrogen) for 10 min at 37 °C. Cells were then washed and mounted as described above.

### Confocal Imaging

Confocal images were captured with a Leica SP5 TCS laser scanning confocal microscope attached to a DM16000 inverted microscope, equipped with a ×63 plan-apochromatic 1.4 NA oil-immersion objective (Leica Microsystems) using the LAS AF Leica software version 1.8.2. All images were captured at 1024 resolution and 100 Hz. Optical slice thicknesses of either 1.074 µm or 0.733 µm are indicated in the figure legends.

The GFP and YFP-tagged constructs were imaged using the 488 nm and the 514 nm excitation bands of an Argon laser. Images were collected at 493–550 nm or 520–600 nm emission bandwidths, respectively. For visualization of the cell nuclei, DAPI was excited with the UV 405 nm laser and images were collected using 410–480 nm emission spectra. Visualization of plasma membranes stained with CellMask™ Deep Red was obtained by exciting the dye with the HeNe633 laser (emission spectra 640–700 nm). The endogenous caveolin1 stained with AlexaFluor555-conjugated goat anti-rabbit antibody, the actin filaments stained with AlexaFluor568-conjugated phalloidin and the structures stained with AlexaFluor555-conjugated WGA were visualized using the HeNe543 laser with an emission bandwidth between 573 and 630 nm.

### Image and Statistical Analysis

ImageJ analysis to determine colocalization coefficient (*Rr*) was performed as previously described (Ayling et al. 2012). Pearson’s coefficient ranges from − 1 to 1, where − 1 represents perfect non-colocalization, 0 means that the signal is entirely random and 1 suggests that the images colocalize perfectly. The signal from each fluorophore was collected sequentially with appropriate controls in order to eliminate bleed-through; the images captured are not saturated. For analysis, the selection was outlined using a polygon selection and the outside cleared for both images to ensure that only the cell of interest is analysed.

Statistical significance of colocalization (*Rr*), densitometry (arbitrary units; AU) and real-time FRET measurements were determined by GraphPad Prism software using Student’s *t* tests with Welch’s correction. In each case, the number in brackets (*n*) refers to the number of cells measured in at least three separate experiments, or the number of times the experiment was repeated, as appropriate. Data are presented as the mean ± standard error of the mean (SEM) or mean ± SE (as indicated) with significance set at *p* < 0.05, where *p < 0.05; **p < 0.01; ***p < 0.001, ****p < 0.0001.

## Results

### Colocalization of Caveolin1 and AC8 in Lipid Rafts Depends on the Cytoskeleton

Previous studies showed that AC8 both co-sediments with caveolin1 on sucrose density gradients following cold detergent extraction (Smith et al. 2002) and also appears to bind to actin microfilaments at the plasma membrane (Ayling et al. 2012). Thus, given the known interaction between caveolin and the cytoskeleton (Muriel et al. 2011), we hypothesized that cytoskeletal disruption might affect the colocalization of AC8 and caveolin1.

**Fig. 1:**
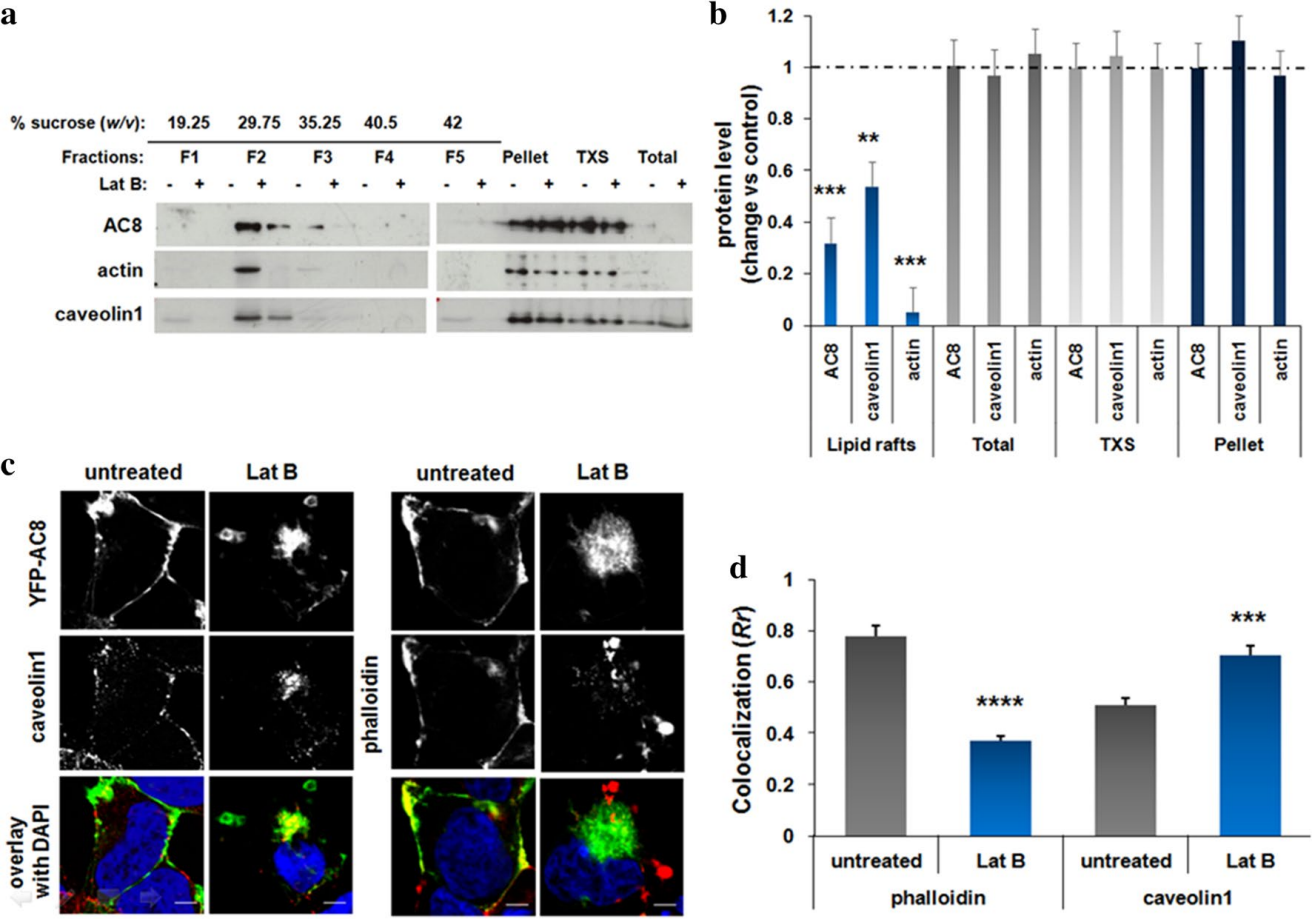
Dissolving the actin cytoskeleton modulates the colocalization between AC8 and caveolin1 **a** HEK293 cells stably expressing YFP-AC8 were homogenized in lysis buffer with 1% Triton X-100. The Triton X-100 soluble (TXS) and insoluble fractions were isolated, and 20 µg of the extracts was analysed by Western blotting. **b** Densitometric analysis (AU) of blots expressed as the ratio of protein after Lat B (2 µM, 1 h, 37 °C) treatment over control (DMSO). Data are presented as mean ± SE (*n* = 3). **c** Representative confocal imaging of HEK293 cells expressing YFP-AC8 treated or not with Lat B and stained with phalloidin (*n* = 18–20) or caveolin1 antibody (*n* = 13–15; scale bars represent 10 µm; optical section thickness = 1.073 µm). **d** Colocalization coefficient (*Rr*) of **c**. Data are presented as mean ± SEM

HEK293 cells stably expressing YFP-tagged AC8 were incubated with Lat B, a macrolide that binds to G-actin and causes passive cytoskeletal depolymerization by sequestering actin monomers and inhibiting ATP/ADP exchange on actin (Spector et al. 1989; Yarmola et al. 2000). Following the treatment, cells were lysed with 1% Triton X-100 and lipid raft fractions were isolated from the soluble extract following sucrose density ultracentrifugation. The protein-to-detergent ratio used in this procedure allows the detection of subtle changes in the lipid raft fraction, while changes in the soluble fractions are unlikely to be detected (Delint-Ramirez et al. 2015). Five equal-volume fractions and the pellet were collected and analysed side-by-side with the soluble extract by immunoblotting (Fig. 1a). As expected, AC8 and caveolin1 were enriched in the lipid rafts fraction. Remarkably, a pool of actin also concentrated in these rafts. Actin, caveolin1 and AC8 were also detected in the soluble extract and the pellet (Fig. 1a). Upon dissolving the cytoskeleton with Lat B, the levels of actin in the lipid raft fractions were significantly reduced. Under these conditions, the levels of AC8 and caveolin1 were also decreased in lipid rafts. The levels of proteins analysed in the soluble extract or pellet were not detectably altered by these treatments (Fig. 1a, b).

Previous reports suggested that AC8 remains associated with actin despite cytoskeletal disruption (Ayling et al. 2012). In the present study, imaging experiments confirmed that in intact cells, AC8 along with F-actin stained with phalloidin colocalized at the plasma membrane (Fig. 1c). To resolve the degree of colocalization, Pearson’s coefficient (*Rr*) was determined as described previously (Ayling et al. 2012). Since our study is assessing a functional relationship between two proteins, Pearson’s coefficient is the most appropriate metric to do so. Pearson’s is in fact superior to other colocalization metrics (Adler and Parmryd 2010) and is currently one of the most extensively used metric to determine colocalization on confocal images (Pike et al. 2017, Aaron et al. 2018). At rest, AC8 displayed a high degree of colocalization with the phalloidin (F-actin) signal (*Rr* = 0.75, Fig. 1c, d). The endogenous caveolin1 detected with a specific antibody presented as two distinct pools, one at the plasma membrane and one intracellular, forming micro-aggregates (Fig. 1c); as had also been observed by Pol et al. (2005). As earlier observed by Ayling et al. (2012), cytoskeletal disruption caused the actin microfilaments to collapse and AC8 aggregated into a cytosolic region proximal to the cell nucleus showing less colocalization with the actin microfilaments than in intact cells (*Rr* = 0.37; Fig. 1c, d). When the distribution of caveolin1 was examined under these conditions, it was seen that caveolin1 internalized and colocalized with AC8 to a greater extent (*Rr* = 0.70) than in resting cells (*Rr* = 0.51; Fig. 1c, d). Thus, these data suggest that cytoskeletal re-arrangements modulate the co-distribution of AC8 with caveolin1 and their recruitment to lipid rafts.

### Knock-Down of Caveolin1 Alters the Behaviour of AC8 on Sucrose Gradients and Its Responsiveness to SOCE

The functional interaction between AC8 and caveolin1 was explored by knock-down of caveolin1 using siRNA. HEK293 cells stably expressing YFP-AC8 were incubated with either caveolin1 siRNA or control (scrambled) siRNA for 24 h. Densitometric analysis determined that caveolin1-selective siRNA-reduced caveolin1 levels in HEK293 cells by approximately 40% compared to control siRNA. The levels of expression of AC8 and actin were not affected by the caveolin1 siRNA knock-down (Fig. 1Sa).

Caveolin1 is regarded as a general kinase inhibitor (Razani et al. 2002). And previous reports suggest that caveolins negatively regulate cAMP signalling (Allen et al. 2009; Sato et al. 2012). To determine a possible functional impact of caveolin1 knock-down on AC8 activity, real-time measurements of cAMP using the Förster resonance energy transfer (FRET)-based cAMP sensor Epac2-camps were performed following caveolin1 siRNA interference. To exploit the strict dependence of AC8 on SOCE channels, SOCE was triggered pharmacologically via passive irreversible depletion of Ca^2+^ stores by addition of thapsigargin (*T*_g_—a sarcoplasmic/endoplasmic reticulum Ca^2+^ATPase inhibitor) followed by addition of [Ca^2+^]_ex_ as previously described (Ayling et al. 2012). Cells transfected with control siRNA displayed a significant cAMP response to SOCE; this response was significantly increased in cells that had been transfected with caveolin1 siRNA (Fig. 1Sb, c). Thus, it appears that caveolin1 exerts a negative influence on AC8 activity.

**Fig. 2:**
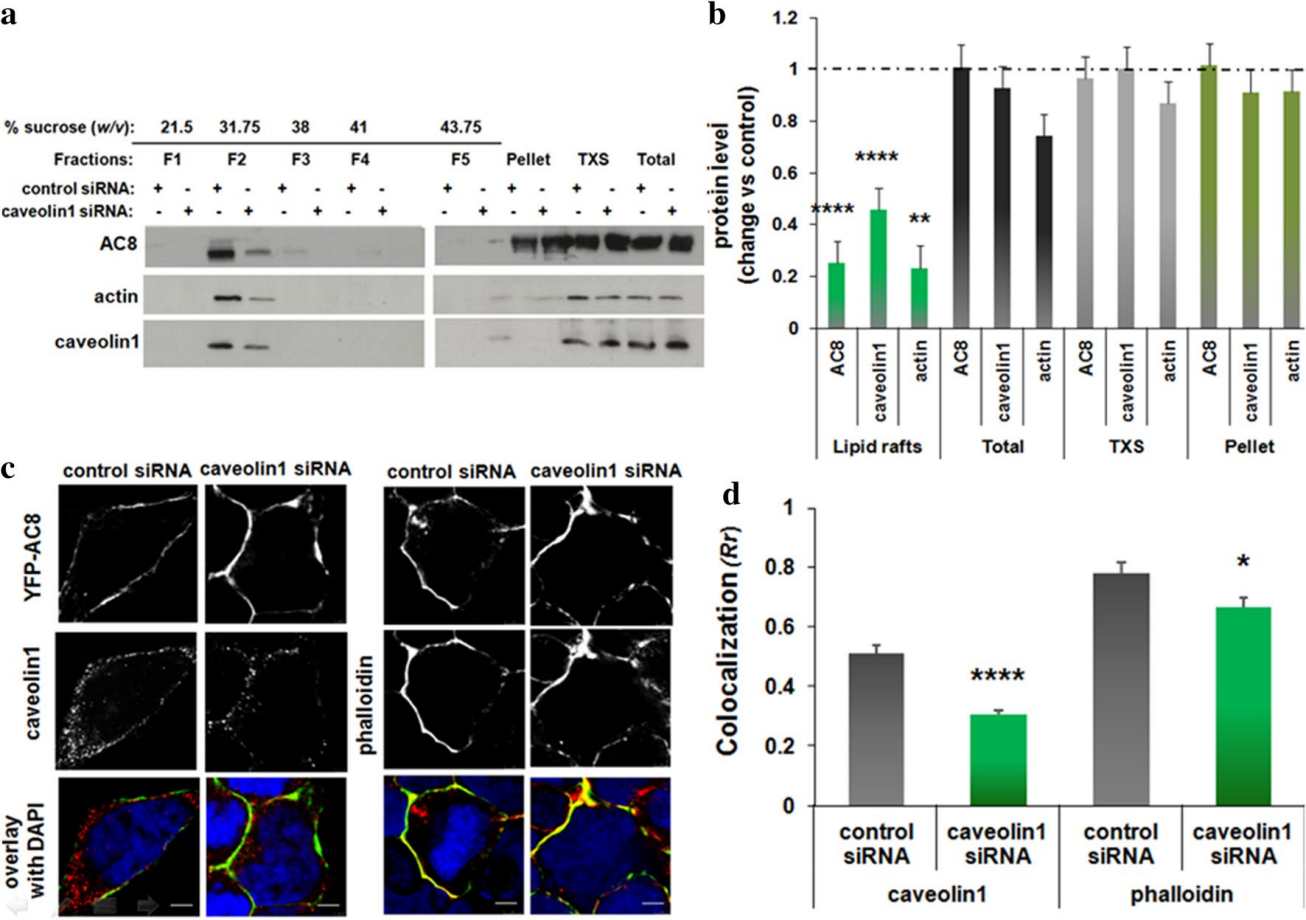
Knock-down of caveolin1 alters AC8 distribution **a** HEK293 cells stably expressing YFP-AC8 were homogenized in lysis buffer with 1% Triton X-100 following transfection of either caveolin1 siRNA (30 nM) or control siRNA (30 nM) for 24 h. The Triton X-100 soluble (TXS) and insoluble fractions were isolated, and 20 µg of the extracts was analysed by SDS-PAGE. **b** Densitometric analysis (AU) of blots expressed as the ratio of protein after knock-down of caveolin1 over control. Data are presented as mean ± SE (*n* = 3). **c** Confocal imaging of HEK293 cells expressing YFP-AC8 transfected with either caveolin1 siRNA or control siRNA and stained with phalloidin (*n* = 25–30) or caveolin1 antibody (*n* = 19–24; scale bars represent 10 µm; optical section thickness = 1.073 µm). **d** Colocalization coefficient (*Rr*) of **c**. Data are presented as mean ± SEM

Next, AC8 distribution following caveolin1 knock-down was assessed by cold detergent extraction as described above. Knock-down of caveolin1 affected the integrity of lipid rafts. Under these conditions, a subtle decrease in AC8 immunoreactivity in the lipid rafts fractions was observed which was mirrored by actin and caveolin1 immunoreactivity (Fig. 2a, b). In parallel, imaging data revealed that neither AC8 nor actin changed distribution in cells in which caveolin1 was knocked down. However, a significant decrease in AC8 colocalization with caveolin1 was detected (*Rr* = 0.30; Fig. 2c, d). Taken together, these data can be taken to indicate that caveolin1 maintains the integrity of the AC8 microenvironment and can act as a negative regulator of SOCE-mediated AC8 activity.

### Mutations in the Caveolin-Binding Sites Impair AC8 N-Linked Glycosylation, Its Plasma Membrane Targeting and Functionality

The persistent functional impact of caveolin1 knock-down on AC8 organization and responsiveness prompted us to search for and investigate residues in the AC8 sequence that might mediate caveolar recruitment. Many proteins that reside in caveolae display ‘caveolin-binding motifs’ (CBM) which are 8–11 hydrophobic polyaromatic stretches with loose, yet identifiable sequences. These sequences are viewed to mediate caveolar recruitment through direct interactions with the CSD (Couet et al. 1997).

**Fig. 3:**
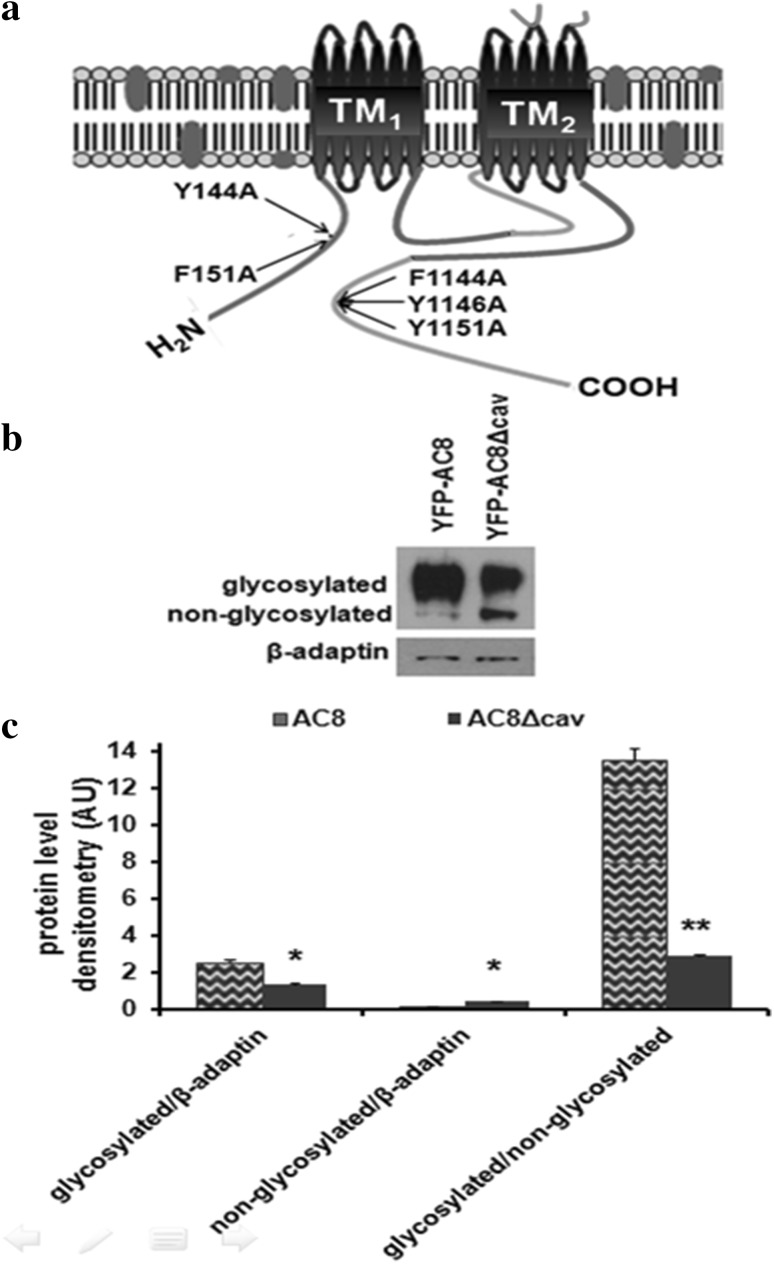
Expression pattern of YFP-AC8Δcav **a** Schematic representation of YFP-AC8Δcav (mutations indicated by arrows). **b** Western blot analysis of crude membranes from HEK293 cells expressing YFP-AC8Δcav. **c** Densitometric analysis (AU) of **b** (*n* = 4). Data are presented as mean ± SEM

Two putative CBM were identified in AC8. The CBMs located at the Nt (residues 144–151) displayed the sequence, YSYRGVIF and at the Ct (residues 1144–1151) the sequence, FDYRGEIY. These CBMs conform to the following sequence ΦXΦXXXXΦ (where X is any amino acid and Φ is an aromatic residue). Within the CBM, it is the aromatic residues that have been demonstrated to mediate the interaction with caveolin proteins (Couet et al. 1997). By site-directed mutagenesis, point mutations in the aromatic residues in the CBM motifs of AC8 were introduced to generate AC8^Y144A, F151A, F1144A, Y1146A, Y1151A^; we shall refer to this construct as YFP-AC8Δcav hereafter (Fig. 3a).

In order to determine how YFP-AC8Δcav was expressed, the construct was transfected into HEK293 cells and lysates were analysed by immunoblotting. Over-expression of YFP-AC8Δcav yielded two immunoreactive bands, which represented the glycosylated (more diffuse band) and the non-glycosylated species (a sharper, lower molecular weight band; Fig. 3b). Indeed, native (transfected) AC8 migrates as two individual species with a more prominent diffuse band running at 165 kDa and a lower molecular weight specie running at 125 kDa corresponding to the glycosylated and non-glycosylated species respectively (Pagano et al. 2009). Compared with native AC8 in the case of YFP-AC8Δcav, the band representing the non-glycosylated species was a lot more prominent than the glycosylated species (Fig. 3b). Quantification of the immunoreactive bands corresponding to YFP-AC8 and YFP-AC8Δcav species by scanning densitometry determined that YFP-AC8Δcav was statistically less glycosylated compared to YFP-AC8 (Fig. 3c).

In order to determine whether these mutations affect the targeting of the AC8, HEK293 cells stably expressing YFP-AC8Δcav were analysed by confocal microscopy. Live-cell imaging indicated that a pool of YFP-AC8Δcav did not overlay with the CellMask™ fluorescent signal, which suggested that at least partly, YFP-AC8Δcav was not confined to the plasma membrane. This observation was also confirmed by determining the colocalization coefficient (*Rr* = 0.49; Fig. 2Sa, b). These findings imply that the CBM may play an important role in the distribution of AC8 at the plasma membrane.

**Fig. 4:**
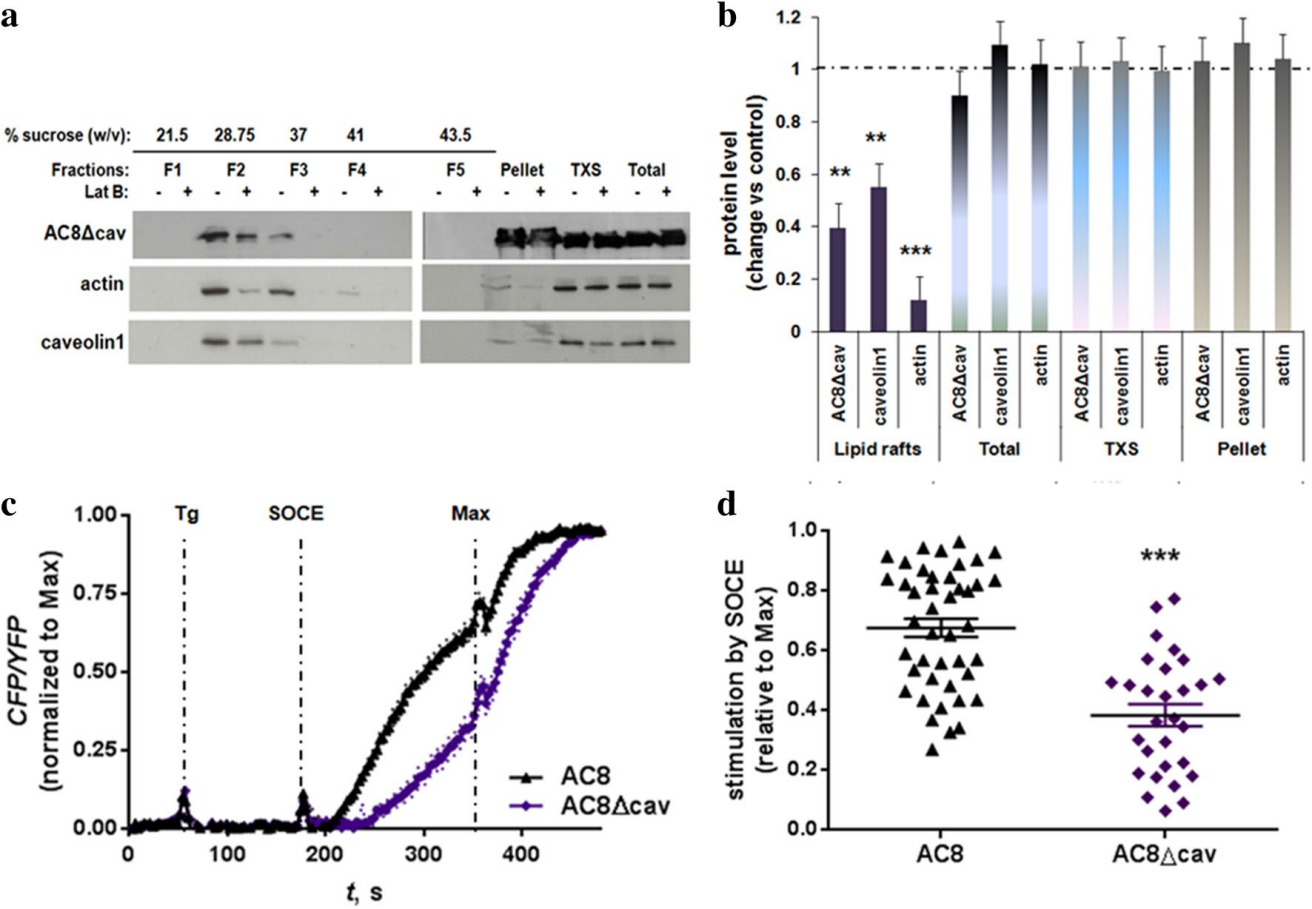
Mutations in the CBM alter AC8 responsiveness **a** HEK293 cells stably expressing YFP-AC8Δcav were homogenized in lysis buffer with 1% Triton X-100. The Triton X-100 soluble (TXS) and insoluble fractions were isolated, and 20 µg of the extracts was analysed by SDS-PAGE. **b** Densitometric analysis (AU) of blots expressed as the ratio of protein after Lat B (2 µM, 1 h, 37 °C) treatment over control (DMSO). Data are presented as mean ± SE (*n* = 3). **c** Single-cell Epac2-camps detection of cAMP in AC8 and AC8Δcav cells following SOCE. Maximum saturation (Max) was attained by addition of a cocktail of Forskolin (10 µM) and IBMX (100 µM) at 360 sec (indicated by arrows). **d** Stimulation by SOCE (relative to Max). Results are plotted as % of Max. Data are presented as mean ± SEM

Given the dissimilarities in terms of the glycosylation profile between AC8 and AC8Δcav, it seemed appropriate to determine whether the mutations in the CBM perturb AC8 organization in lipid rafts. Therefore, lipid raft fractionation of HEK293 cells stably expressing YFP-AC8Δcav was performed before and after treatment with Lat B as previously described. Unexpectedly, in resting cells, YFP-AC8Δcav co-immunopurified along with caveolin1 on sucrose gradients (Fig. 4a). Moreover, just like in the case of native YFP-AC8 (Fig. 1), disrupting the actin microfilaments lowered the affinity of YFP-AC8Δcav for lipid rafts (Fig. 4a, b). Next, we assessed the impact of the CBM mutations on AC8 functionality by measuring the cAMP production following SOCE in HEK293 cells expressing AC8Δcav. Real-time measurements of cAMP using Epac2-camps revealed that by statistically robust criteria, AC8Δcav was approximately 40% less responsive to SOCE than full-length AC8. At the same time, a delay in the responsiveness of AC8Δcav was recorded (Fig. 4c, d). Taken together, these data imply the CBM may not be involved in the recruitment of AC8 to caveolae. However, given that the mutations affected AC8 responsiveness to SOCE, our data suggest that other mechanisms, besides the necessity of residence in lipid rafts may be required for full AC8 functionality.

### N-Linked Glycosylation Targets AC8 to the Plasma Membrane but not to Lipid Rafts

**Fig. 5:**
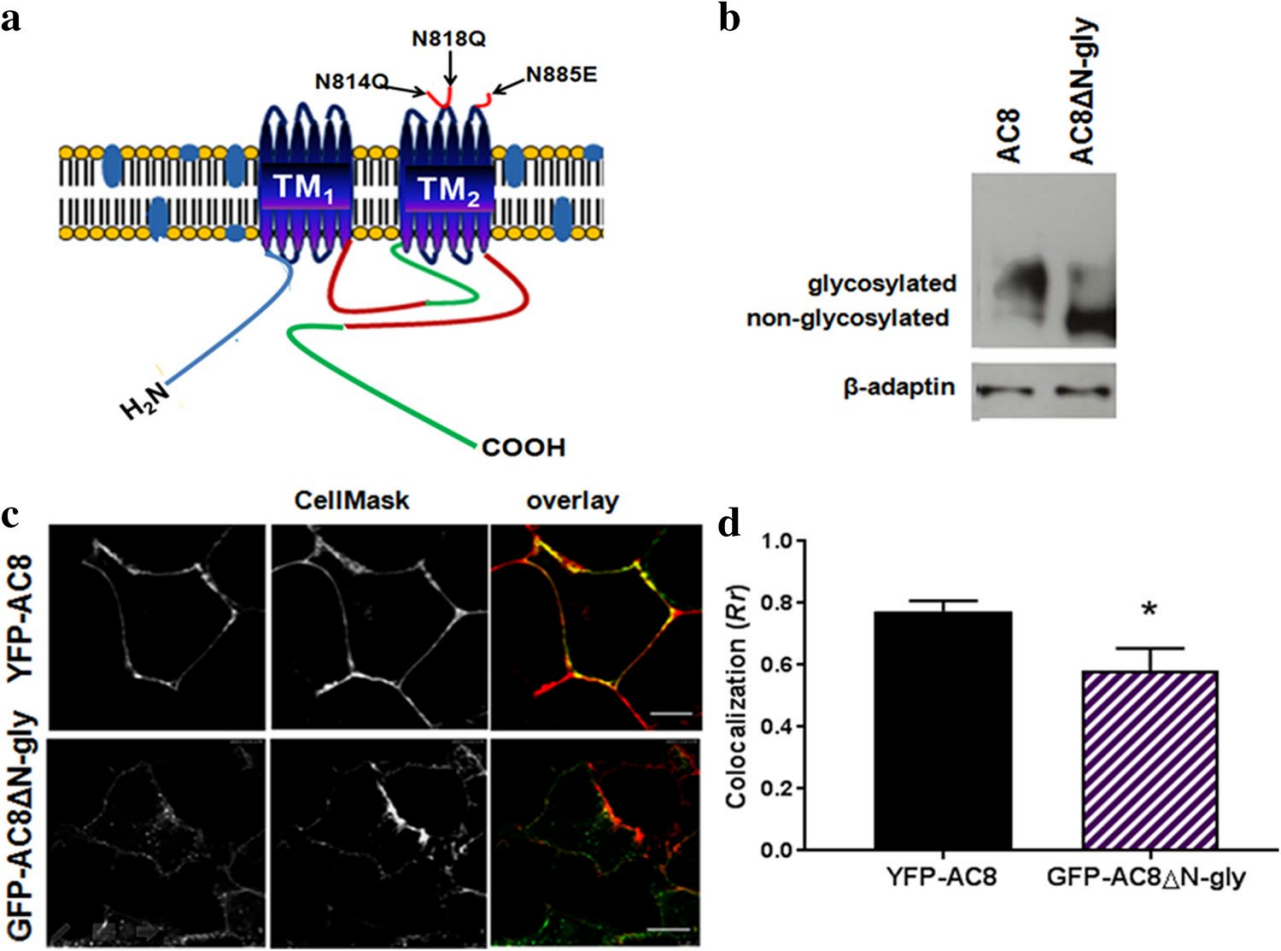
N-linked glycosylation directs AC8 to the plasma membrane **a** Schematic representation of AC8 depicting its predicted topology. AC8-Nt is shown in blue, C1a and C2a domains are in red, C1b and C2b fragments are shown in green. The three asparagine residues that constitute potential N-linked glycosylation sites located on the 5th and 6th extracellular loops within the second TM casette (shown by black arrows) were mutated to glutamine (N814 and N818) and glutamic acid (N885) to generate AC8ΔN-gly. **b** Western blot analysis of crude membranes from HEK293 cells stably expressing AC8 and AC8ΔN-gly (6% acrylamide gels) probed for AC8 and β-adaptin by immunoblotting. **c** Confocal imaging of AC8 and AC8ΔN-gly in overlay with CellMask™ Deep Red (*n* = 25–30; scale bars represent 10 µm; optical section thickness = 0.773 µm). **d** Colocalization analysis (*Rr*) of **c**. Data are presented as mean ± SEM. (Color figure online)

Since AC8Δcav displayed altered N-linked glycosylation profiles, targeting and activity, we next investigated the likely link between N-linked glycosylation and AC8 functionality. For this purpose, we used a mutant of AC8, in which the three putative N-glycosylation asparagine residues were mutated to generate GFP-AC8^N814Q,N818Q,N855E^, described formerly by Pagano et al. (2009) and termed hereafter AC8ΔN-gly, in which N-linked glycosylation was precluded (Fig. 5a).

As previously determined, stable expression of AC8ΔN-gly yielded a single immunoreactive band on Western blots, corresponding to the non-glycosylated AC8 (Fig. 5b). Stably expressed AC8ΔN-gly largely, but not entirely, exhibited a plasma membrane-like distribution. Careful colocalization studies determined that the AC8ΔN-gly fluorescent signal showed substantially less overlay with the plasma membrane fluorescent marker, CellMask™ (*Rr* = 0.58) compared to full-length AC8 (*Rr* = 0.77; Fig. 5c, d). These results imply that glycosylation plays a partial role in trafficking AC8 to the plasma membrane. Time-lapse confocal imaging revealed that transiently transfected full-length AC8 distributed at the plasma membrane within approximately 48-h post-transfection; an event which paralleled its N-linked glycosylation (Fig. 3Sa, b). These data suggest that N-linked glycosylation and AC8 confinement at the plasma membrane are likely to be interconnected events.

**Fig. 6:**
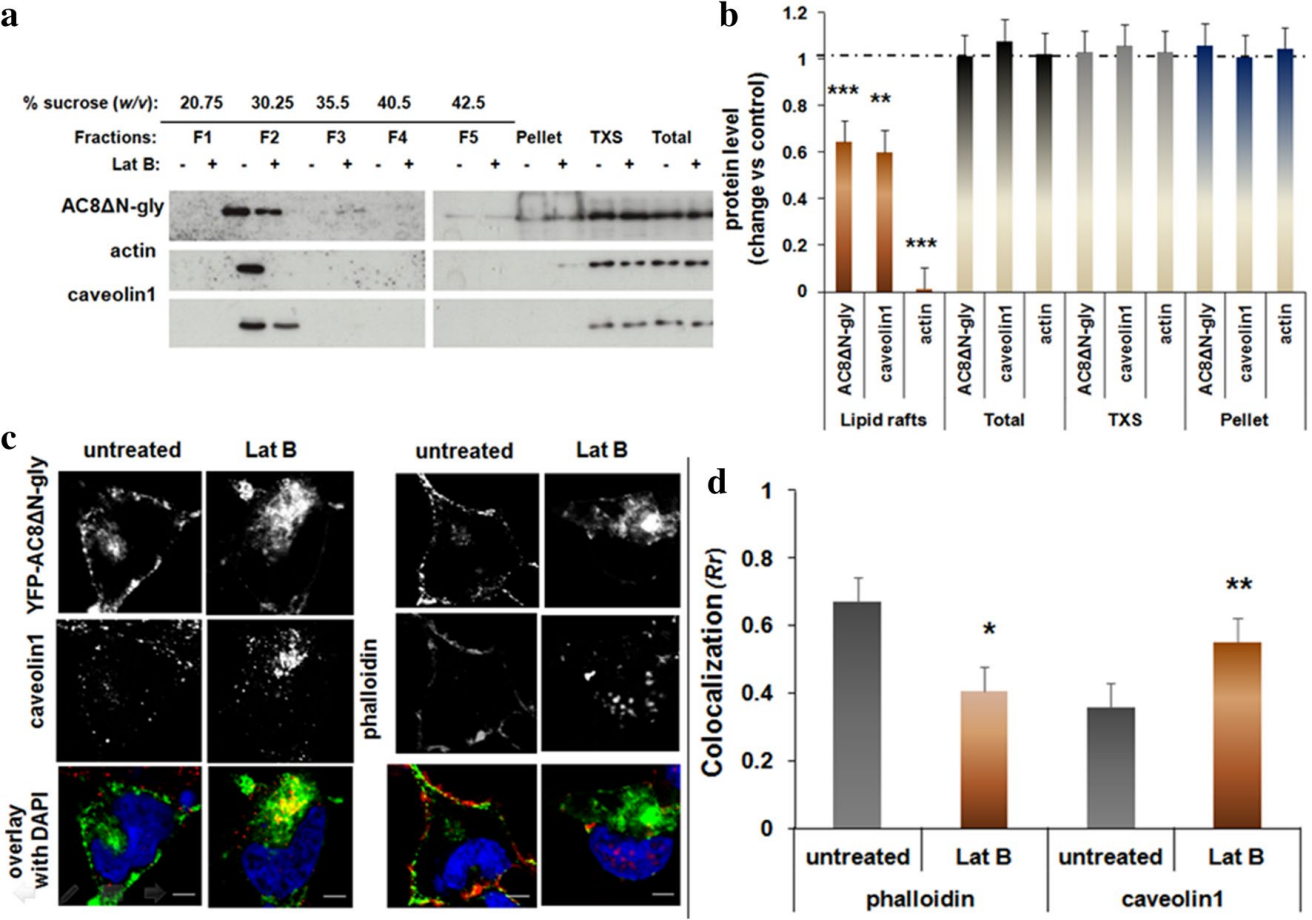
AC8ΔN-gly enriches in lipid rafts **a** HEK293 cells stably expressing GFP-AC8ΔN-gly were homogenized in lysis buffer with 1% Triton X-100. The Triton X-100 soluble (TXS) and insoluble fractions were isolated, and 20 µg of the extracts was analysed by SDS-PAGE. **b** Densitometric analysis (AU) of blots expressed as the ratio of protein after Lat B (2 µM, 1 h, 37 °C) treatment over control (DMSO). Data are presented as mean ± SE (*n* = 3). **c** Confocal imaging of HEK293 cells expressing GFP-AC8ΔN-gly treated or not with Lat B and stained with phalloidin (*n* = 10–13) or caveolin1 antibody (*n* = 12–15; scale bars represent 10 µm; optical section thickness = 1.073 µm). **d** Colocalization analysis (*Rr*) of **c**. Data are presented as mean ± SEM

Transiently transfected AC8ΔN-gly was localized outside lipid rafts (Pagano et al. 2009). However, the residence of AC8 in lipid rafts is necessary for its regulation by SOCE (Smith et al. 2002). Consequently, AC8ΔN-gly was stably expressed and fractionated on sucrose gradients. Lipid raft isolation demonstrated that, unlike transiently transfected AC8ΔN-gly, stably expressed AC8ΔN-gly co-fractionated with caveolin1 and actin on sucrose gradients (Fig. 6a, b). The relative distribution of GFP-AC8ΔN-gly and the actin cytoskeleton was then analysed by confocal imaging. GFP-AC8ΔN-gly showed considerable overlay with the actin signal (*Rr* = 0.67), albeit to a lesser extent than full-length AC8, which indicates the existence of a significantly larger intracellular pool due to compromised targeting. Treatment with Lat B internalized AC8ΔN-gly, which still maintained a degree of association with the actin signal (*Rr* = 0.40). Furthermore, under these conditions, AC8ΔN-gly aggregated and colocalized with caveolin1, mirroring the behaviour of AC8 (*Rr* = 0.55; Fig. 6c, d).

To further verify the association of AC8ΔN-gly with lipid rafts, cholesterol was extracted with MβCD, a water-soluble cyclic oligosaccharide which encloses a hydrophobic core acting as a carrier for amphipathic molecules such as cholesterol. Due to its cholesterol-binding properties, MβCD has been broadly used to deplete cells of cholesterol (Zidovetzki and Levitan 2007). Treatment with MβCD resulted in extrusion of AC8ΔN-gly and caveolin1 and an increase in their colocalization coefficient (*Rr* = 0.62; Fig. 4Sa). These data indicate that GFP- AC8ΔN-gly largely resides in lipid rafts.

Earlier single time-point measurements of cAMP in populations of cells suggested that AC8ΔN-gly and AC8 display similar activation profiles. Slight, statistically insignificant differences in their responsiveness were attributed to dissimilarities in their levels of expression (Pagano et al. 2009). Given the inherent limitations in the spatio-temporal resolution of such measurements in populations of cells, we measured cAMP in single cells as a function of time using Epac2-camps. These real-time measurements revealed that AC8ΔN-gly was less responsive statistically to SOCE than AC8 (Fig. 4Sb, c).

Summarized, these data point towards a role of N-glycosylation in appropriate AC8 distribution at the plasma membrane, which is of course a pre-requisite for its functionality. Thus, residence in lipid rafts although essential, does not entirely account for AC8 responsiveness to SOCE. Other factors such as plasma membrane targeting may contribute to AC functionality.

### AC8 is Glycosylated with Hybrid or Complex Glycans and Acquires Detergent Insolubility as It Travels Through Golgi

Lipid rafts are proposed to form during transition via the Golgi through the association of cholesterol with sphingolipids (Brown and Rose 1992; Simons and Toomre 2000). In order to determine whether AC8 acquires detergent insolubility upon transiting through the Golgi we used BFA, a fungal metabolite which blocks the anterograde ER to Golgi transport and the secretory flux inside the Golgi (Donaldson et al. 1992; Klausner et al. 1992).

**Fig. 7:**
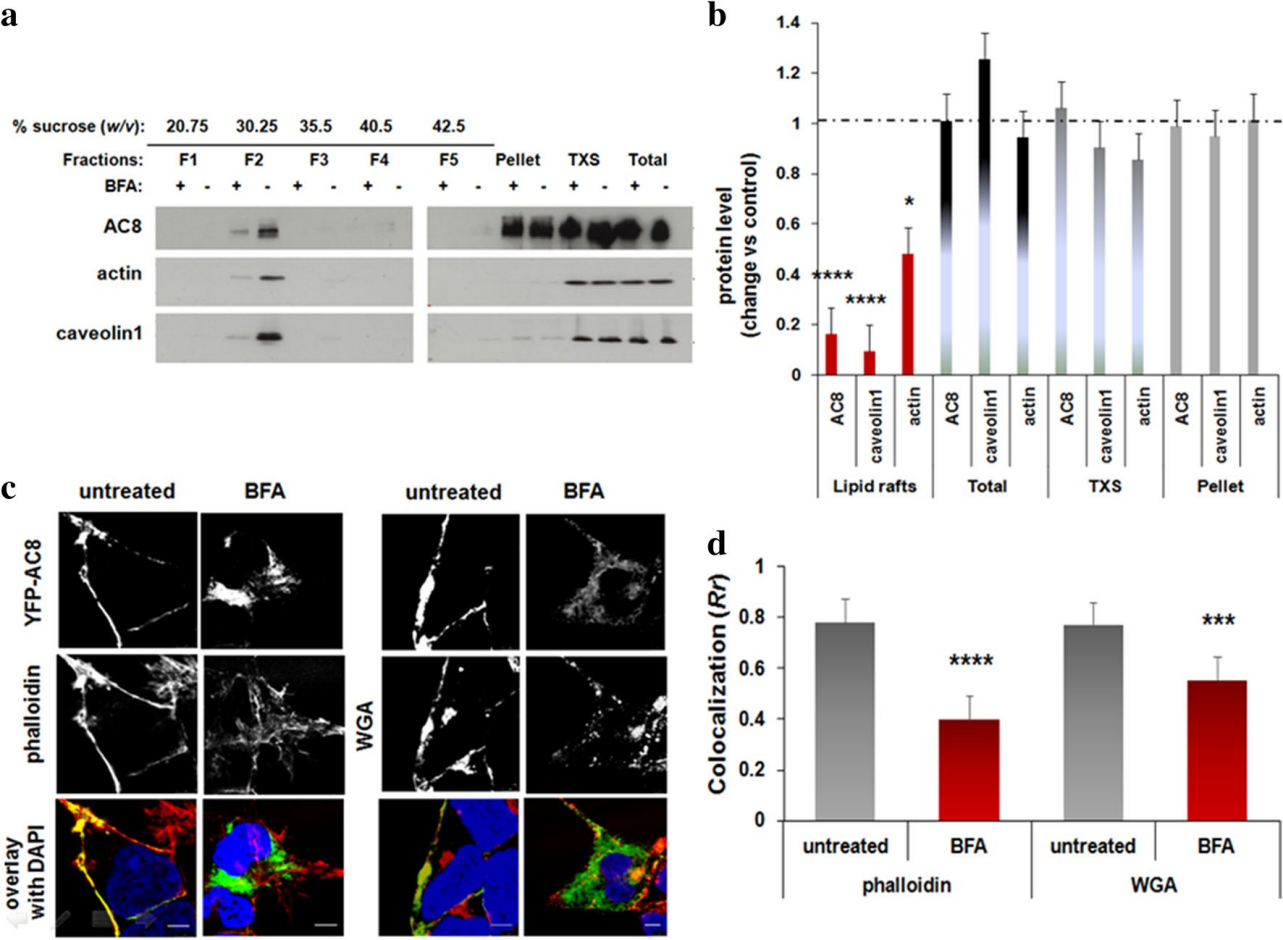
Blockage of ER to Golgi trafficking decreases the association of AC8 with lipid rafts **a** HEK293 cells stably expressing YFP-AC8 were homogenized in lysis buffer with 1% Triton X-100. The Triton X-100 soluble (TXS) and insoluble fractions were isolated, and 20 µg of the extracts was analysed by SDS-PAGE. **b** Densitometric analysis (AU) of blots expressed as the ratio of protein after BFA (5 µg/ml) treatment over control (DMSO). Data are presented as mean ± SE (*n* = 3). **c** Confocal imaging of HEK293 cells expressing YFP-AC8 treated or not with BFA (5 µg/ml) and stained with phalloidin or WGA (scale bars represent 10 µm; optical section thickness = 1.073 µm). **d** Colocalization coefficient (*Rr*) of **c**. Data are presented as mean ± SEM

Following treatment with BFA and extraction with Triton X-100, a pool of AC8 and caveolin1 shifted from the Triton X insoluble fractions to the soluble fractions on sucrose gradients (Fig. 7a, b). Solubilization of AC8 and caveolin1 may be explained by their entrapment in the ER due to blockage of ER to Golgi transport by BFA. Remarkably, treatment with BFA caused a pool of actin to dissociate from the buoyant fractions (Fig. 7a, b). At the same time, the AC8 fluorescent signal re-located from the plasma membrane towards the cell interior, as determined by confocal imaging. At rest, the microfilaments appeared condensed at the plasma membrane; however, chronic treatment with BFA caused the cytoskeleton to collapse and aggregate towards the cell cytosol, adopting a more widespread appearance, perhaps due to de-polymerization (Fig. 7c). AC8 and actin signals, although partly overlapping, showed less colocalization than in resting cells (*Rr* = 0.39; Fig. 7d).

In order to better visualize the relative distribution of AC8 following treatment with BFA, cells were stained with wheat germ agglutinin (WGA). This lectin specifically binds sialic acid and *N*-acetyl-d-glucosamine contained in glycoproteins and glycolipids, making it an excellent option for staining plasma membrane and the Golgi (Chazotte 2011). In resting cells, AC8 showed a high degree of colocalization with the WGA signal at the plasma membrane (*Rr* = 0.76; Fig. 7c, d). Chronic treatment with BFA caused the WGA signal to relocate from the plasma membrane and the Golgi into a widespread structure, the ER (Fig. 7c). In parallel, AC8 also redistributed from the plasma membrane displaying partial overlay with the WGA signal (*Rr* = 0.55; Fig. 7d).

A time course of BFA treatment revealed that the drug impaired AC8 glycosylation to some extent but did not affect its dimerization (Fig. 5Sa, b). Typically, glycoproteins are modified with high mannose N-glycans co-translationally in the ER and they may be further modified with hybrid and complex N-glycans in the cis- and medial compartments of the Golgi (Vagin et al. 2009). Since BFA inhibits the ER to Golgi trafficking as well as the AC8 glycosylation pattern, our data suggest that AC8 is processed with hybrid and/or complex glycans. Thus, blockage of ER to Golgi trafficking impairs AC8 glycosylation, the assembly of AC8 microdomain and the integrity of the cytoskeleton.

## Discussion

The essential residence of ACs at the plasma membrane is a neglected topic. Obviously for ACs to be regulated by G protein coupled receptor-based mechanisms or even by the elements of SOCE, the AC must encounter these regulators at the plasma membrane. However, what is not known and what is definitely understudied is how AC dwells at the plasma membrane and the influences that promote its proper targeting to the points where it will be regulated. In this study, we have used a number of cellular approaches to attempt to both consolidate and expand upon the various tacks that have emerged from previous studies of ACs or of other surface plasma membrane proteins. In this regard, we have considered that a dynamic interplay might pertain between a range of contributory elements. For instance, we know that the AC explored in the study, AC8, must necessarily reside in lipid rafts for its regulation by SOCE (Smith et al. 2002). Cholesterol-binding domains are sometimes a means for prolonging the associations of such raft targeted proteins (Lorent and Levental 2015). We also know that the AC, like many plasma membrane proteins, must be properly N-glycosylated to be targeted to the plasma membrane (Gu et al. 2001; Vagin et al. 2009). Additionally, AC8 is found in association with caveolin1 (Figs. 1, 2). Again, we also know from earlier studies that AC8 undergoes essential associations with the actin cytoskeleton (Ayling et al. 2012). Consequently, here we have taken a comprehensive, dynamic approach where we try to consolidate these various influences into a comprehensive integrated sense of the trafficking and delivery of the AC to and from the plasma membrane.

We have made a number of observations of multiple factors contributing to the proper targeting of the AC, which when organized contextually allow a picture to emerge of a trafficking process that can be interrogated at various points and which allows a clear sense of flow to emerge. Very simply, AC8 binds both actin and caveolin1. At the same time, the cytoskeleton and caveolin1 are critical determinants of the integrity of lipid rafts (Cohen et al. 2004; Head et al. 2014). From knock-down of caveolin1 and disruption of the cytoskeleton with Lat B, both caveolin1 and the cytoskeleton are seen to be crucial for maintaining the integrity of the AC8 microdomain (Figs. 1, 2). Additionally, the integrity of lipid rafts is required for proper AC8 functionality. Interestingly, dissociation of AC8 from lipid rafts following caveolin1 knock-down hyperactivates the enzyme (Fig. 1Sb, c). This result is consistent with previous findings assigning caveolins a role in tonic inhibition of AC (Toya et al. 1998; Allen et al. 2009).

During secretion from the Golgi, AC8, like many plasma membrane-targeted proteins (Vagin et al. 2009), is essentially N-glycosylated with hybrid and/or complex glycans (Fig. 7, 5Sa, b); the pattern of glycosylation dictates its association with the plasma membrane and its functionality (Fig. 5, 4Sb, c). Interestingly, residence of AC8 in lipid rafts does not seem to depend on its glycosylation status: both AC8Δcav and AC8ΔN-gly remained associated with lipid rafts despite obvious defects in glycosylation and responsiveness (Figs. 4, 6). Critically, AC8 becomes an integral part of lipid rafts prior to its arrival to the plasma membrane (Fig. 7).

AC8 displays caveolin-binding motifs (CBM), which is a common means by which many raft-resident proteins are believed to be targeted to lipid rafts (Byrne et al. 2012). Mutation of these CBM disrupts proper post-translational processing (N-linked glycosylation) of AC8 (Fig. 3) and consequently negatively affects both its targeting to the plasma membrane and its responsiveness (Fig. 4c, d, 2Sa, b).

Our imaging experiments support colocalization of AC8 with caveolin1—the best documented and widely accepted *bona fide* raft proxy, which was used to assess the quality of our biochemical preparation. Data collected following knock-down of caveolin1 represents further indirect evidence for AC8-caveolin1 association. These data alongside the identification of caveolin1-binding motifs in the primary structure of AC8 and the link of their mutation to AC8 processing and trafficking represent a strong body of evidence for the presence of AC8 in lipid rafts. Thus, in summary, the data gathered by our biochemical approach are supported by high-resolution confocal microscopy, mutagenesis, pharmacological tools and FRET experiments as well as the responsiveness of AC8 to physiological stimuli in addition to previously published data.

Based on these explorations, we envisage a model for AC8 processing, trafficking, assembly into cholesterol-rich domains and targeting to the plasma membrane lipid rafts. We conclude that AC8 traffics to and from the plasma membrane in a cyclical process that involves synthesis in the ER, progression through the Golgi where it is further N-glycosylated. Somewhere along the secretory pathway, AC8 becomes associated with caveolin1 and is targeted to internal rafts which themselves traffic to and from the plasma membrane (Simons and Ikonen 1997; Cooper and Tabbasum 2014). In addition, caveolin1 may control AC8 functionality at the plasma membrane either directly or indirectly via interactions with AC8. Thus, caveolin1 exerts complex effects on AC8 processing, trafficking and responsiveness. This model, within the context of trafficking and processing of membrane proteins, offers an efficient explanation for the complex assembly and organization of AC8 in lipid rafts.

The realization of the range of the potential AC–caveolin interactions raises further questions. Resolving the details of such interactions may help to better understand the dynamics of AC movement and its integration in lipid rafts. From the current data, we cannot conclude whether caveolin1 and AC8 bind via a direct or an indirect interaction. Hence, future studies should be directed towards understanding the specific mechanisms underlying these interactions and their contribution to the secretion of ACs, their targeting and functionality in lipid rafts.

## Electronic supplementary material

Below is the link to the electronic supplementary material.

**Fig. 1S** Knock-down of caveolin1 alters AC8 functionality **a** HEK293 cells stably expressing YFP-AC8 were transfected with either caveolin1 siRNA (30nM) or control siRNA (30nM) for 24h, lysed and resolved by 12% polyacrylamide SDS-PAGE and probed for AC8, actin and caveolin1 (left panel). Densitometric analysis (AU) of **a** (n=3) (right panel). Results are presented as the mean +/- S.E.M. **b** Single cell Epac2-camps detection of cAMP in AC8 cells transfected with either caveolin1 siRNA or control siRNA following SOCE. Maximum saturation (Max) was attained by addition of a cocktail of Forskolin (10μM) and IBMX (100μM) at 300s (indicated by arrows). Symbols represent means, error bars, S.E.M. **c** Stimulation by SOCE (relative to Max). Results are plotted as % of Max. Data are presented as mean +/- S.E.M. Supplementary material 1 (TIF 179 KB)

**Fig. 2S** Distribution of YFP-AC8Δcav **a** Confocal imaging of HEK293 cells expressing YFP-AC8 and YFP-AC8Δcav in overlay with CellMask^TM^ Deep Red (n=21-26; scale bars represent 10μm; optical section thickness=1.074μm). **b** Colocalization analysis (*Rr*) of **a**. Data are presented as mean +/- S.E.M. Supplementary material 2 (TIF 173 KB)

**Fig. 3S** Time-lapse visualization of AC8 trafficking **a** Time-lapse visualization of transiently-transfected YFP-AC8 in overlay with DAPI by confocal imaging (n=6; scale bars represent 5μm; optical section thickness=0.733μm). **b** Western blot analysis of crude membranes from HEK293 cells transiently expressing AC8-HA (10μg protein per lane; 7% polyacrylamide gel) probed for AC8 and β-adaptin. Supplementary material 3 (PNG 102 KB)

**Fig. 4S** Lack of N-linked glycosylation alters AC8 responsiveness **a** left panel - confocal imaging of HEK293 cells expressing GFP-AC8ΔN-gly treated or not with MβCD (10mM, 1h, 37°C) and immunostained with caveolin1 antibody (n=9-12; scale bars represent 10μm; optical section thickness=1.073μm); right panel – colocalization coefficient (*Rr*). **b** Single cell Epac2-camps detection of cAMP in HEK293 cells expressing AC8 and AC8ΔN-gly following SOCE. Maximum saturation (Max) was attained by addition of a cocktail of Forskolin (10μM) and IBMX (100μM) at 420s (indicated by arrows). **c** Stimulation by SOCE (relative to Max). Results are plotted as % of Max. Data are presented as mean +/- S.E.M. Supplementary material 4 (PNG 149 KB)

**Fig. 5S** Treatment with BFA alters AC8 N-linked glycosylation **a** HEK293 cells stably expressing AC8-HA were treated with BFA for the indicated periods of time, lysed and resolved by 7% acrylamide SDS-PAGE and probed for AC8 and β-adaptin by immunoblotting. **b** Densitometry (AU) of **a**. Data are presented as mean +/- S.E.M. (n=4). Supplementary material 5 (PNG 113 KB)
