# Supplementary figures and images for: Structural and Functional Determinants of AC8 Trafficking, Targeting and Responsiveness in Lipid Raft Microdomains
Source: J Membr Biol. 2019 Feb 12;252(2):159–72. doi: 10.1007/s00232-019-00060-x (PMC6556161; doi:10.1007/s00232-019-00060-x)

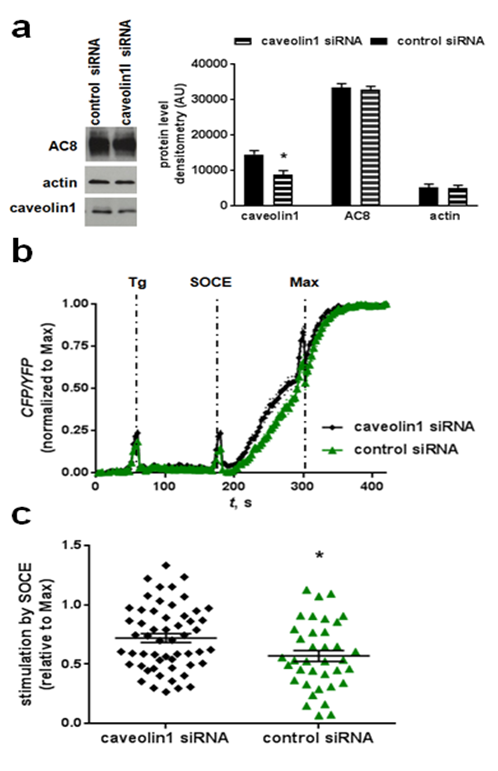

Supplement: Supplementary file 1 — Fig. 1S Knock-down of caveolin1 alters AC8 functionality a HEK293 cells stably expressing YFP-AC8 were transfected with either caveolin1 siRNA (30nM) or control siRNA (30nM) for 24h, lysed and resolved by 12% polyacrylamide SDS-PAGE and probed for AC8, actin and caveolin1 (left panel). Densitometric analysis (AU) of a (n=3) (right panel). Results are presented as the mean +/- S.E.M. b Single cell Epac2-camps detection of cAMP in AC8 cells transfected with either caveolin1 siRNA or control siRNA following SOCE. Maximum saturation (Max) was attained by addition of a cocktail of Forskolin (10μM) and IBMX (100μM) at 300s (indicated by arrows). Symbols represent means, error bars, S.E.M. c Stimulation by SOCE (relative to Max). Results are plotted as % of Max. Data are presented as mean +/- S.E.M. Supplementary material 1 (TIF 179 KB) [file 232_2019_60_MOESM1_ESM.tif]

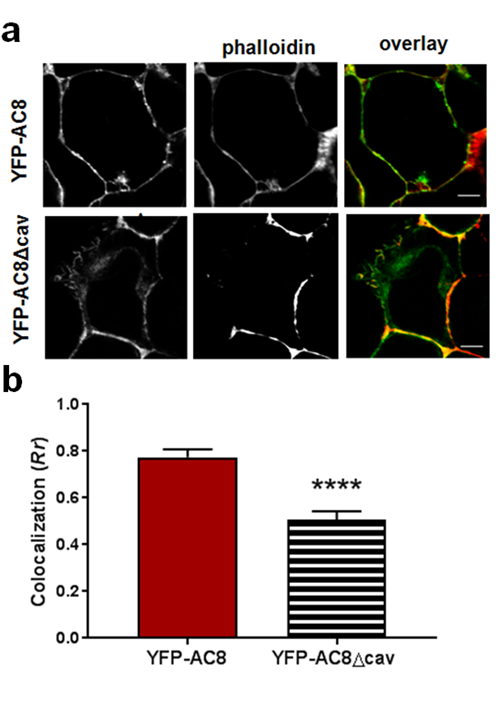

Supplement: Supplementary file 2 — Fig. 2S Distribution of YFP-AC8Δcav a Confocal imaging of HEK293 cells expressing YFP-AC8 and YFP-AC8Δcav in overlay with CellMaskTM Deep Red (n=21-26; scale bars represent 10μm; optical section thickness=1.074μm). b Colocalization analysis (Rr) of a. Data are presented as mean +/- S.E.M. Supplementary material 2 (TIF 173 KB) [file 232_2019_60_MOESM2_ESM.tif]

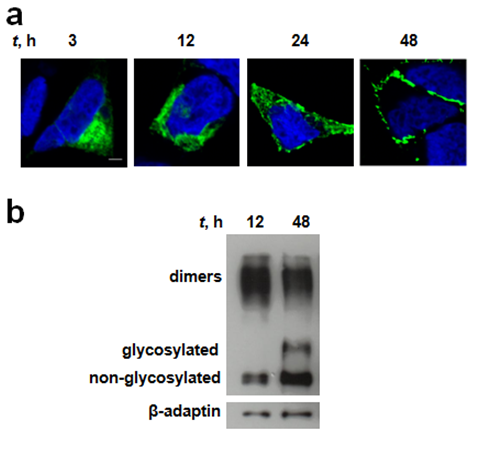

Supplement: Supplementary file 3 — Fig. 3S Time-lapse visualization of AC8 trafficking a Time-lapse visualization of transiently-transfected YFP-AC8 in overlay with DAPI by confocal imaging (n=6; scale bars represent 5μm; optical section thickness=0.733μm). b Western blot analysis of crude membranes from HEK293 cells transiently expressing AC8-HA (10μg protein per lane; 7% polyacrylamide gel) probed for AC8 and β-adaptin. Supplementary material 3 (PNG 102 KB) [file 232_2019_60_MOESM3_ESM.png]

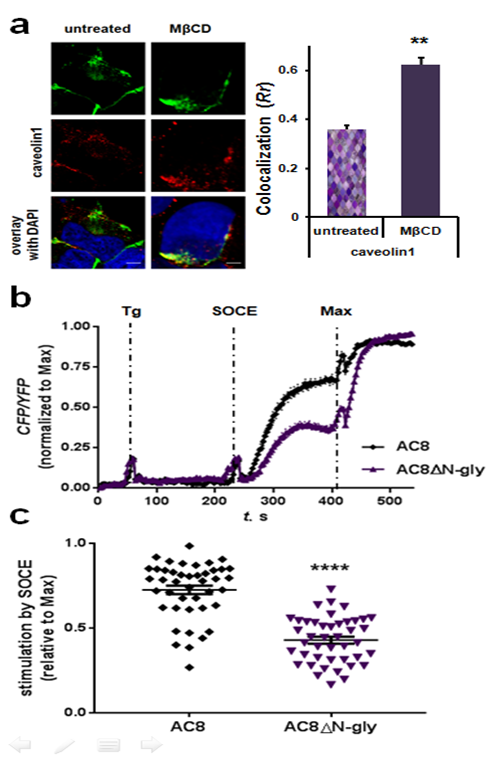

Supplement: Supplementary file 4 — Fig. 4S Lack of N-linked glycosylation alters AC8 responsiveness a left panel - confocal imaging of HEK293 cells expressing GFP-AC8ΔN-gly treated or not with MβCD (10mM, 1h, 37°C) and immunostained with caveolin1 antibody (n=9-12; scale bars represent 10μm; optical section thickness=1.073μm); right panel – colocalization coefficient (Rr). b Single cell Epac2-camps detection of cAMP in HEK293 cells expressing AC8 and AC8ΔN-gly following SOCE. Maximum saturation (Max) was attained by addition of a cocktail of Forskolin (10μM) and IBMX (100μM) at 420s (indicated by arrows). c Stimulation by SOCE (relative to Max). Results are plotted as % of Max. Data are presented as mean +/- S.E.M. Supplementary material 4 (PNG 149 KB) [file 232_2019_60_MOESM4_ESM.png]

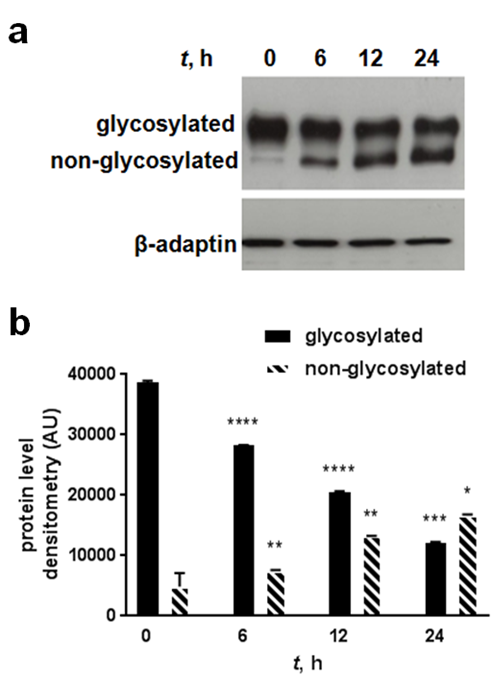

Supplement: Supplementary file 5 — Fig. 5S Treatment with BFA alters AC8 N-linked glycosylation a HEK293 cells stably expressing AC8-HA were treated with BFA for the indicated periods of time, lysed and resolved by 7% acrylamide SDS-PAGE and probed for AC8 and β-adaptin by immunoblotting. b Densitometry (AU) of a. Data are presented as mean +/- S.E.M. (n=4). Supplementary material 5 (PNG 113 KB) [file 232_2019_60_MOESM5_ESM.png]
